# RETRACTED: Rahimifard et al. Therapeutic Effects of Gallic Acid in Regulating Senescence and Diabetes; an In Vitro Study. *Molecules* 2020, *25*, 5875

**DOI:** 10.3390/molecules30173542

**Published:** 2025-08-29

**Authors:** Mahban Rahimifard, Maryam Baeeri, Haji Bahadar, Shermineh Moini-Nodeh, Madiha Khalid, Hamed Haghi-Aminjan, Hossein Mohammadian, Mohammad Abdollahi

**Affiliations:** 1 Toxicology and Diseases Group, Pharmaceutical Sciences Research Center (PSRC), The Institute of Pharmaceutical Sciences (TIPS), Tehran University of Medical Sciences, Tehran 1417613151, Iranmadihakhalid777@gmail.com (M.K.);; 2 Institute of Paramedical Sciences, Khyber Medical University, Peshawar 25120, Pakistan; 3 Pharmaceutical Sciences Research Center, Ardabil University of Medical Sciences, Ardabil 5618953141, Iran; 4 Department of Toxicology and Pharmacology, School of Pharmacy, Tehran University of Medical Sciences, Tehran 1417614411, Iran

The journal retracts the article, “Therapeutic Effects of Gallic Acid in Regulating Senescence and Diabetes; an In Vitro Study” [1] cited above.

Following publication, concerns were brought to the attention of the publisher regarding image irregularities contained within this article [1].

Adhering to our standard procedure, an investigation was conducted that confirmed the duplication, with indication of inappropriate editing, between Figure 2A,C in [1] and between Figure 2D in [1] and Figure 4D in [2], produced by a similar authorship group presenting different experimental conditions. Given the nature of these concerns, the Editorial Board has lost confidence in the validity of the overall findings and has decided to retract this article [1] as per MDPI’s retraction policy (https://www.mdpi.com/ethics#_bookmark30).

This retraction was approved by the Editor-in-Chief of the journal *Molecules*.

Mohammad Abdollahi did not agree to this retraction. The remaining authors did not provide a comment on this decision.
